# The role of SARS1 in the progression of colorectal cancer: insights from clinical data and organoid models

**DOI:** 10.7717/peerj.21792

**Published:** 2026-10-25

**Authors:** Ayimukedisi Yalikong, Jing-yi Liu, Bahejuan Jiaerken, Dong-li He, Zhang-han Chen, Zhi-peng Qi, Qiang Shi, Yun-shi Zhong

**Affiliations:** 1 Endoscopy Center and Endoscopy Research Institute, Zhongshan Hospital, Fudan University, Shanghai, China; 2 Endoscopy Center, Xuhui Hospital, Zhongshan Hospital of Fudan University, Shanghai, China

**Keywords:** Colorectal adenoma organoid, Colorectal cancer, Serine tRNA ligase, Biomarker

## Abstract

Colorectal cancer (CRC) is a significant global health concern, with adenomas representing critical precancerous lesions. Early detection of adenomas is essential for reducing the incidence of CRC; however, effective biomarkers for the adenoma–carcinoma transition are currently lacking. Serine tRNA ligase (SARS1) has been identified in colorectal adenoma tissues, but its biological role remains unclear. Here, we analyzed SARS1 expression in colorectal adenoma and colorectal cancer using data from the GEO and TCGA databases, as well as immunohistochemical staining of 48 tissue pairs (normal colorectal mucosa, adenoma lesion, and cancer lesion). SARS1 expression was significantly higher in adenomas and cancers compared to normal colorectal mucosa, decreasing from the adenoma to cancer stages. High SARS1 expression was associated with improved overall survival (OS; Log-rank *P* = 0.0209) and progression-free survival (PFS; Log-rank *P* = 0.0147) in patients with CRC. As demonstrated by immunofluorescence and qRT-PCR, in colorectal adenoma organoids, SARS1 knockdown was associated with decreased expression of LGR5 and OLFM4, and increased expression of c-Myc. This gene expression profile mirrors that observed in colorectal cancer organoids, as reported in previous studies. Additionally, the downregulation of SARS1 also enhanced the proliferation of CRC cells and the viability of colorectal adenoma organoids (*p* < 0.01 to *p* < 0.0001). Our findings suggest that SARS1 exerts context-dependent, stage-associated growth-suppressive effects during the adenoma-to-carcinoma transition, and its downregulation is associated with colorectal cancer progression. SARS1 represents a candidate marker reflecting adenoma-to-carcinoma progression; however, its clinical utility as a diagnostic or prognostic tool requires independent validation in larger, independent, adequately powered cohorts, and its precise functional mechanisms warrant further investigation.

## Introduction

Cancer development is a complex multistage process, evolving from precancerous lesions to malignant tumors (Rosty et al., 2013). Colorectal cancer (CRC) ranks as the third most common cancer and the second leading cause of cancer-related deaths globally (Bray et al., 2024). Its development typically commences with adenomas or serrated lesions (Rosty et al., 2013). Approximately 85% of CRCs originate from adenomas, with low-grade and high-grade intraepithelial neoplasia being critical precancerous lesions (Xiong et al., 2025). Thus, the “normal colorectal epithelial mucosa–adenoma–carcinoma” sequence represents the classic model of CRC development. During the precancerous stages, the accumulation of genetic alterations and abnormal protein expression drives the gradual abnormal development of tissues, ultimately progressing to adenocarcinoma (De Palma et al., 2019). Early detection of adenomas and their removal *via* endoscopy can effectively reduce the incidence of CRC and improve survival rates, underscoring the significance of early detection and resection of colorectal adenomas (Duvvuri et al., 2021; Lin et al., 2021; Heisser et al., 2023). However, due to the low concentration of biomarkers in precancerous lesions, there is currently a paucity of effective early diagnostic indicators and markers for accurately assessing the risk of adenoma–carcinoma transition (Cai et al., 2021; Xiong et al., 2025). Therefore, research on biomarkers for colorectal adenomas is not only crucial for early diagnosis but also for dynamically tracking lesion progression through changes in biomarkers, enabling the timely detection of exacerbated dysplasia or signs of cancer transformation. Investigating the expression and regulatory patterns of biomarkers in colorectal precancerous lesions and cancer can help elucidate the transformation process from benign to malignant lesions and the pathogenesis of CRC (Sun et al., 2024).

Previous studies have delved into the key molecular mechanisms of colorectal adenoma carcinogenesis by analyzing the transcriptomic profiles of normal tissue, adenomas, and cancer tissues from patients with CRC (Luo et al., 2023; Xiong et al., 2025). However, the screening and validation of candidate biomarkers based on various omics data necessitate extensive bioinformatics analysis. Previously, we enriched and captured serine tRNA ligase (SerRS or SARS1) from colorectal adenoma tissue lysates using the MORAC method, which relies on affinity and catalytic molecular recognition (Hu, 2024). We then employed a DNAzyme-based method to validate the expression levels of SARS1 in a limited number of samples (Hu, 2024). Our findings revealed that SARS1 expression was 10 times higher in adenomas compared to normal colorectal mucosa and 2 times higher in cancers compared to normal colorectal mucosa (Hu, 2024). However, we did not delve into the specific biological functions of SARS1 in colorectal adenomas and cancers.

SARS1 is a classical aminoacyl-tRNA synthetase, primarily tasked with linking serine to its corresponding tRNA to participate in protein synthesis (Liu et al., 2023). However, during evolution, SARS1 acquires multiple non-canonical functions, particularly in transcriptional regulation within the cell nucleus (Xu et al., 2012). SARS1 can enter the nucleus *via* its unique carboxyl-terminal domain (UNE-S) and act as a transcriptional repressor to modulate gene expression, such as in the transcriptional regulation of vascular endothelial growth factor A (VEGFA) (Xu et al., 2012). VEGFA is a factor implicated in angiogenesis and is crucial for cancer development (Song et al., 2019; Zou et al., 2020). Additionally, SARS1 can function as a transcription factor to inhibit tumor growth, a role that has been demonstrated in breast cancer and prostate cancer (Zou et al., 2020; Zhang et al., 2020). However, the role of SARS1 in colorectal adenomas and cancer remains enigmatic. Thus, this study aims to explore the biological functions of SARS1 in the progression from normal colorectal tissue to adenoma to cancer and to investigate its potential as a candidate marker associated with the malignant transformation of colorectal adenomas and cancer.

## Materials & Methods

### Tissue samples

A total of 144 tissue samples, including 48 pairs of normal, adenoma, and carcinoma tissues from 48 patients, were collected at Zhongshan Hospital of Fudan University between November 2021 and August 2022. For each patient, matched normal mucosa, adenoma, and carcinoma tissues were obtained simultaneously, enabling paired comparisons. Pathological diagnoses were confirmed by two independent pathologists with expertise in CRC. Ethical approval for this study was obtained from the Ethics Committee of Zhongshan Hospital of Fudan University (B2021-777R). And, we have received written informed consent from participants of our study.

### Construction and passage of colorectal adenoma organoids

Colorectal adenoma organoids were established from (*n* = 3) independent patient-derived adenoma samples. Each organoid line was derived from a distinct patient and cultured separately to ensure biological independence. Fresh colorectal adenoma tissue samples were rinsed with Epithelial Organoid Basal Medium (Serum-free) (B213151; bioGenous BIOTECH, Inc., Suzhou, China) until the supernatant was clear before being minced into small fragments and digested using Tumor Tissue Digestion Solution (K601003; bioGenous) in SmartOrgan Dissociator (bioGenous)/at 37 °C for 20∼30 min. The digestion was terminated by adding 2–4% fetal bovine serum. The supernatant was collected, passed through a 100 µm strainer, and centrifuged. The erythrocytes were lysed with Red Blood Cell Lysis Solution (E238010, bioGenous) and centrifuged. The cell pellet was suspended in extracellular matrix (ECM) (M315066; bioGenous) and plated into 24-well plates. The organoids were overlaid with Human Colorectal Adenoma Organoid Medium (K2102-CR; bioGenous). The medium was changed every 2 days. The organoids were passaged using Organoid Dissociation Solution (E238001; bioGenous) or cryopreserved using Organoid Cryopreservation Medium (E238023; bioGenous).

### Histology and immunohistochemistry

Samples were fixed with 4% paraformaldehyde for 1 h at room temperature. The samples were then embedded in paraffin and sliced to a thickness of 8 µm. All sections were dried at 60 °C for 120 min and incubated in xylene before being dehydrated in alcohol. The sections were either stained with hematoxylin and eosin (H&E) or processed for immunofluorescence (IF). After immunohistochemical staining, the staining results were quantitatively analyzed using the H-Score method (H-Score=∑(pi×i)=(percentage of weak intensity×1)+(percentage of moderate intensity×2)+(percentage of strong intensity×3)) (Dogan et al., 2019; Maclean et al., 2020). The data and immunohistochemistry images were provided in File S3.

### Immunofluorescent staining

For organoids, the procedure was conducted as previously described by Shi et al. (2022). IF staining was performed after incubation in 0.1 mol/L citrate buffer (pH 6.0) in a boiling water bath for 20 min, and nonspecific binding was further blocked using 5% goat serum for 60 min (Shi et al., 2022). The sections were incubated with the primary antibody overnight at 4 °C and then washed (Shi et al., 2022). This was followed by Alexa-Fluor-488 and Alexa-Fluor-555-conjugated goat secondary antibodies against mouse, goat, or rabbit (1:1,000; Invitrogen (Thermo Fisher Scientific, Waltham, MA, USA)). Nuclei were counterstained with 4’,6-diamidino-2-phenylindole (DAPI) (Shi et al., 2022). The detailed antibody information is shown in Table S4. All primary antibodies used in this study are listed in Table S4, including supplier name, catalog number, clone/host species, and working dilution. Quantitative IF analysis was carried out by calculating relative fluorescence intensity from three independent experiments using ImageJ (Fiji; https://imagej.net/software/fiji/) v1.53, with background subtraction and threshold-based segmentation (Files S4 and S5). Experiments were conducted on organoids derived from (n = 3) independent patients (one organoid line per patient). Owing to limited sample yields, technical replicates were not performed; instead, biological replication across three distinct patient backgrounds was employed to ensure generalizability.

### Cell line and culture protocol

HCT-116 cells were purchased from the Shanghai Cell Bank of the Chinese Academy of Sciences. The cells were cultured in Dulbecco’s Modified Eagle Medium (DMEM) supplemented with 10% fetal bovine serum at 37 °C in a 5% CO_2_ atmosphere. DLD-1 and SW480 cells were purchased from Wuhan Servicebio Technology Co., Ltd. (Wuhan, China). The cells were cultured under the same conditions. All cell lines were routinely tested for mycoplasma contamination using the MycAway™ Plus-Color One-Step Mycoplasma Detection Kit (40612ES; Yeasen Biotechnology, Shanghai, China) to ensure they were free of mycoplasma infection.

### SARS1 knockdown in Colorectal adenoma organoids and colorectal cancer cells

#### shRNA design

The three validation sequences of SARS1 genes were selected by logging into the Sigma shRNA design platform, and three shRNA targeting sequences were obtained by using the reverse complementary sequences on the SMS2 Nanjing DETA biological mirror website. The target sequence and primer sequence are shown in Tables S1 and S2, respectively.

#### Plasmid construction and extraction

The pLKO.1 TRC-cloning vector and helper plasmids were retrieved from an ultra-low-temperature refrigerator for extended culture, and the plasmids were extracted using endotoxin-free kits (DP118-02; Tiangen Biotech, Beijing, China). An amount of 6 µg plasmid was digested with AgeI (R3552S; New England Biolabs (NEB), Ipswich, MA, USA) and EcoRI (R3101V; NEB) at 37 °C overnight. The linearized 7 Kb fragment was gel-purified using a FastPure Gel DNA Extraction Mini Kit (DC301-01, Vazyme, Nanjing, China) and quantified. Using T4 PNK (M0201V; NEB), the primer was annealed through a thermal cycling procedure consisting of 37 °C for 30 min, followed by 95 °C for 5 min, and then gradual cooling to 25 °C. The T4 DNA ligase (EL0014; Thermo Fisher Scientific) was used to ligate the annealed primer, digest the DNA, and ligate the DNA at 22 °C overnight. Ligation products were transformed into *Escherichia coli* competent cells. Colonies were screened on LB agar plates containing ampicillin (100 µg/mL). Positive clones were validated by Sanger sequencing (U6 universal primers).

#### Plasmid viral transfection

One day before transfection, 293T cells were passaged to achieve a cell density of about 40–50%. DNA and Lipu2000 dilutions (SB-C005; Shanghai ShareBio, Shanghai, China) were prepared, mixed to form a complex, and added to 293T cells. The viral supernatant was collected at 72 h post-transfection, centrifuged (1,500 ×g, 15 min, 4 °C), and concentrated using PEG-it™ Virus Precipitation Solution (HG-LPKC100; HonorGene, Changsha, China). The supernatant was stored at −80 °C.

#### SARS1 overexpression lentivirus

The SARS1 overexpression lentivirus used in this section was constructed and provided by Genechem (Shanghai, China) (Cat. No.: GOSL0401617).

#### Infection and screening

The organoids or cells were digested into clusters. Then, the cells were resuspended in complete medium and viral concentrate and cultured in a 37 °C CO2 incubator for 12 h. Next, the supernatant was removed, the Organoid Culture ECM (M315066; bioGenous) was added and mixed, and the complete medium was added after the matrix gel solidified. The cells were added to the complete medium only. Infected organoids or cells were selected by puromycin (2 µg/mL) for 7 days. Surviving organoids or cells were expanded for downstream analysis.

#### Real-time quantitative PCR

Real-time quantitative PCR (qRT-PCR) conducted by our lab was used to detect the knockdown efficiency of SARS1 and the expression levels of C-MYC, LGR5, OLFM4, and SARS1 in control group (cells or adenoma organoid) and SARS1 knockdown group (cell or adenoma organoid infected with sh-SARS1). For organoids, the sample volume was the amount that fills five wells of a 24-well plate; for cells, it was the amount that fills one well of a six-well plate. The RNA was immediately snap-frozen in liquid nitrogen after extraction and then transferred to a −80 °C freezer for storage. RNA extraction was performed using the Trizol-chloroform method. Organoids were added to Trizol reagent and lysed thoroughly. Chloroform was added, followed by vigorous shaking and centrifugation. The aqueous phase was transferred to a new tube, and isopropanol was added to precipitate the RNA. The RNA was washed with 75% ethanol and dissolved in an appropriate amount of RNAse-free water after drying. The RNA concentration of each sample was determined using the Thermo Fisher Scientific nucleic acid concentration detector. After zeroing, 1 µl of RNA solution was dropped on the testing table for reading. The purity of RNA was assessed by the A260/A280 ratio, which ranged from 1.8 to 2.2. The yield of RNA was between 4–7 µg. DNase treatment was conducted according to the instructions provided in the PrimeScript™ RT Reagent Kit (RR037A; Takara, Kyoto, Japan) manual to eliminate gDNA contamination. Reverse transcription was performed using the PrimeScript™ RT Reagent Kit (RR037A; Takara). The reaction conditions were as follows: 37 °C for 15 min, followed by 85 °C for 5 s. Each reaction contained 500 ng of RNA in a total volume of 10 µL. The remaining cDNA should be stored in a −80 °C freezer. qPCR was performed using the SYBR Green Mix (B21202; Selleckchem, Houston, TX, USA). The complete reaction conditions were according to the kit user’s manual. Each reaction contained 5 ng of cDNA in a total volume of 20 µL, with primer concentrations at 0.5 µM. The thermocycling parameters were as follows: Warming at 95 °C for 10 min, PCR (40 cycles): 95 °C for 15 s, 60 °C for 30 s, 72 °C for 30 s, followed by dissolution curves: 95 °C for 15 s, 60 °C for 60 s, 95 °C for 15 s. The qPCR was conducted using the QuantStudio™ 1 Plus System (Thermo Fisher Scientific). The reaction setup was manual, and each sample was run in triplicate. For the primer sequences, please refer to Table S3. The synthesis of oligonucleotides was provided by Sangon Biotech (Shanghai, China). The amplicon length ranged from 100 to 300 bp. The specificity of the primers was confirmed by Basic Local Alignment Search Tool (BLAST) validation on the National Center for Biotechnology Information (NCBI) database. The specificity of the qPCR products was confirmed by melting curve analysis, with the melting temperature (Tm) and melting peak height checked in File S2. The quantification cycle (Cq) values ranged from 15 to 35, indicating the limit of detection. No modifications were made to the primers, probes, or templates in this study. the plates of the qRT-PCR is the MicroAmp Optical 96-Well Reaction Plate (Applied Biosystems™, N8010560).

The qRT-PCR analysis was performed using the QuantStudio™ 1 Plus System. The threshold for Cq determination was automatically set by the qPCR software to a level that was above the baseline fluorescence but below the exponential phase of the amplification curve. This automatic threshold was validated to ensure consistency across all runs and samples. Outliers were identified based on the quality inspection results of the instrument. If the cycle threshold (Ct) value was too large or the amplification efficiency was low, the amount of template was increased to see if the Cq value could be reduced accordingly. The qPCR reaction conditions were checked to ensure the primer design was reasonable. The results were normalized using the housekeeping gene β-actin. The expression levels of the target genes were normalized to the expression level of β-actin in each sample. The intra-assay variation was assessed with three technical replicates. Statistical analysis was performed using one-way analysis of variance (ANOVA) or t-tests, with GraphPad Prism (version 10.0.3; https://www.graphpad.com/updates/prism-1003-release-notes) used for data analysis. And, the results of qRT-PCR were analyzed using the ΔΔCT method. For organoid experiments, qRT-PCR was performed on (*n* = 3) independent patient-derived adenoma organoid lines per group, with each sample run in triplicate technical replicates. The raw Cq values, melting curve data, and amplification plots are provided in File S2.

### Western Blot

Two groups of cell lines (negative control and SARS1 knockdown or over-expression) were lysed using radioimmunoprecipitation assay (RIPA) Lysis and extraction buffer (P0013B; Beyotime Biotechnology, Shanghai, China) supplemented with 1% protease inhibitor cocktail (P1005; Beyotime) and 1% phosphatase inhibitor cocktail (P1081; Beyotime) on ice for 30 minutes, and then centrifuged at 12,000 ×g for 10 min at 4 °C. The protein samples were subjected to electrophoresis at 80 V for 30 min in a stacking gel, followed by electrophoresis at 110 V for 100 min in a separating gel. The proteins were transferred to a 0.2 µm nitrocellulose membrane (NC; Merck Millipore, Burlington, MA, USA). The membrane was blocked with a 5% skim milk solution for 2 h and then incubated overnight at 4 °C with an anti-human SARS1 polyclonal rabbit antibody (1:2000, 15162-1-AP; Proteintech, Rosemont, IL, USA). The following day, the membrane was washed using Tris-Buffered Saline with Tween-20 (TBST) and incubated for 2 h at 25 °C with an anti-rabbit horseradish peroxidase (HRP)-conjugated secondary antibody (1:2000, SA00001-2; Proteintech). After the reaction, the proteins were visualized using enhanced chemiluminescence (ECL) Western Blotting Substrate (KF8005, Yeasen) and a ChemiCapture system (CLINX; https://clinxsci.com/). For sequential detection of multiple targets, the same membrane was stripped using Beyotime Western Blot Antibody Stripping Buffer (P0025B; Beyotime) and subsequently reprobed with the indicated antibodies. Full-length blot images are presented; note that some nonspecific bands may appear due to prior antibody incubations on the same membrane. Each cell line was subjected to Western blot analysis with 2–3 technical replicates to ensure reproducibility. Western blot images and corresponding molecular weight markers are provided in File S1.

### Organoid living cell viability analysis

Organoids were passaged and dissociated into single cells. Cells were seeded at a density of 5,000 cells per well in 24-well plates for each experimental group, with technical replicates included. Experiments were performed using (*n* = 3) independent patient-derived adenoma organoid lines per group. Organoid growth was monitored using the BioGenous LivingCell-FluoTM Organoid Vitality Assay Kit (E238004; bioGenous) at four time points during the growth cycle: days 4, 7, 9, and 13. Wells without organoids were designated as blank controls. Prior to analysis, the original culture medium was aspirated from the test wells, and a uniformly mixed viability detection working solution was gently added along the sidewalls of all test wells. The plates were then incubated at 37 °C with 5% CO2 for 1 h. Total fluorescence intensity values were measured using a microplate reader. After detection, the working solution was discarded, and a fresh complete organoid culture medium was replenished to resume cultivation until the subsequent monitoring time point. Fluorescence intensity values from every time point were statistically analyzed to generate organoid growth and metabolic viability curves.

To observe the morphology and density of the two groups during growth, organoids were passaged and dissociated into single cells. Cells were seeded at a density of 100,000 cells per well in 10 cm dish plates for each experimental group. Microscopic imaging and observation were performed on Days 0, 4, and 9 after plating. The original data are provided in File S2.

### EdU cell proliferation assay

Cells were seeded at an appropriate density in 12-well plates and cultured until they reached the logarithmic growth phase. Subsequently, a 10 µM 5-ethynyl-2’-deoxyuridine (EdU) labeling solution was added, and the cells were incubated for 2 h. The culture medium was then removed, and the cells were washed with phosphate-buffered saline (PBS). The cells were fixed with 4% paraformaldehyde for 15 min, followed by washing with PBS and permeabilization using 0.5% Triton X-100 for 20 min. The EdU reaction solution (EdU-555, CX003; Epizyme Biomedical Technology, Shanghai, China) was added according to the kit’s instructions and reacted in the dark for 30 min. After washing, the cell nuclei were optionally stained using DAPI or Hoechst. Finally, the cells were observed under a fluorescence microscope, and the percentage of EdU-positive area was analyzed using ImageJ to evaluate cell proliferation.

### Cell Counting Kit-8 Assay

Cells were seeded at a density of 1,000 cells per well in a 96-well plate. After a 24-hour pre-culture, 10 µL of the Cell Counting Kit-8 Assay (CCK-8) solution (40203ES; Yeasen) as added to each well, followed by incubation for 1.5-2 h. Absorbance was measured at 450 nm using a microplate reader. The cell viability or proliferation was assessed by calculating the ratio of absorbance between the experimental and control groups. The original data are provided in File S2.

### Statistical analyses

Statistical analysis was performed using SPSS 29.0.2.0 (IBM Corp., Armonk, NY, USA) or GraphPad Prism 10.0.3. Fisher’s exact probability method or *χ*2 tests were used to evaluate the associations between SARS1 and clinical parameters. A value of *P* < 0.05 was considered statistically significant (two-sided). In the analysis of gene expression data, the significance of differences between two groups of samples was assessed using either the Wilcoxon test or Student’s *t*-test, depending on the distribution of the data. For matched tissue comparisons (normal *vs.* adenoma *vs.* carcinoma from the same patient), paired statistical tests were applied where appropriate, and the paired structure of the data was explicitly accounted for in the analysis. For comparisons involving three groups of samples, analysis of one-way ANOVA was employed to determine the significance of gene expression differences. In the Kaplan–Meier survival analysis, we used the log-rank test to compare the survival differences between patients with high and low SARS1 expressions in colorectal cancer, as obtained from the The Cancer Genome Atlas (TCGA) database. For the Kaplan–Meier survival analysis, the optimal cutoff value for gene expression was determined using the surv_cutpoint() function from the survminer package. This data-driven cutoff selection was performed within the same TCGA cohort, and the resulting survival associations should be interpreted as exploratory rather than definitive prognostic claims. Based on this cutoff value, samples were divided into two groups: high expression and low expression of the SARS1 gene. The significance of survival differences between these groups was assessed using the Log-rank test. Additionally, the hazard ratio (HR) with a 95% confidence interval (CI) was calculated using univariate Cox regression analysis. All analyses were conducted using R software version 4.0.3 (R Core Team, 2020). A *p*-value of less than 0.05 was considered statistically significant. The *R* packages “*survival*” (DOI: 10.32614/CRAN.package.survival) and “survminer” (DOI: 10.32614/CRAN.package.survminer) were used for *survival* analysis.

### Data Availability

All raw data supporting the findings of this study, including uncropped Western blot images (File S1), original immunohistochemistry scans (File S3), qRT-PCR raw Cq values (File S2), original images of IF stain of different genes (File S4 and File S5), and EdU analysis data (File S6) are available in the Supplementary Materials. The raw data for the IF and H&E images for organoid construction and the EdU proliferation assay images are available in Figshare: https://doi.org/10.6084/m9.figshare.34037907 (IF and H&E) and https://doi.org/10.6084/m9.figshare.34037853 (EdU).

## Results

### Relationship of SARS1 expression with the progression of colorectal adenoma and cancer

To elucidate the expression differences in SARS1 between colorectal adenoma and colorectal cancer, we analyzed datasets from the Gene Expression Omnibus (GEO) database (GSE8671) and the TCGA database (consisting of 600 tumor samples and 51 normal colorectal mucosa samples). Additionally, the expression differences in SARS1 were confirmed through immunohistochemical staining and data analysis of 48 pairs of matched colorectal tissue samples (encompassing normal colorectal mucosa, adenomas, and cancers derived from the same patients). Our findings showed that, in comparison with normal tissue, the expression of SARS1 was upregulated in colorectal adenomas and colorectal cancers (*p* < 0.0001, Figs. 1A, 1B). Additionally, the statistical analysis of the H-score from the immunohistochemical staining of 48 pairs of matched clinical samples demonstrated that, compared with normal colorectal mucosa, SARS1 expression was higher in adenomas and cancers (*P* = 0.002 for normal *vs.* CRC; *P* < 0.0001 for normal *vs.* adenoma; Figs. 1C, 1D). Compared with adenomas, SARS1 expression was lower in colorectal cancer (*P* = 0.040; Figs. 1C, 1D).

**Figure 1:**
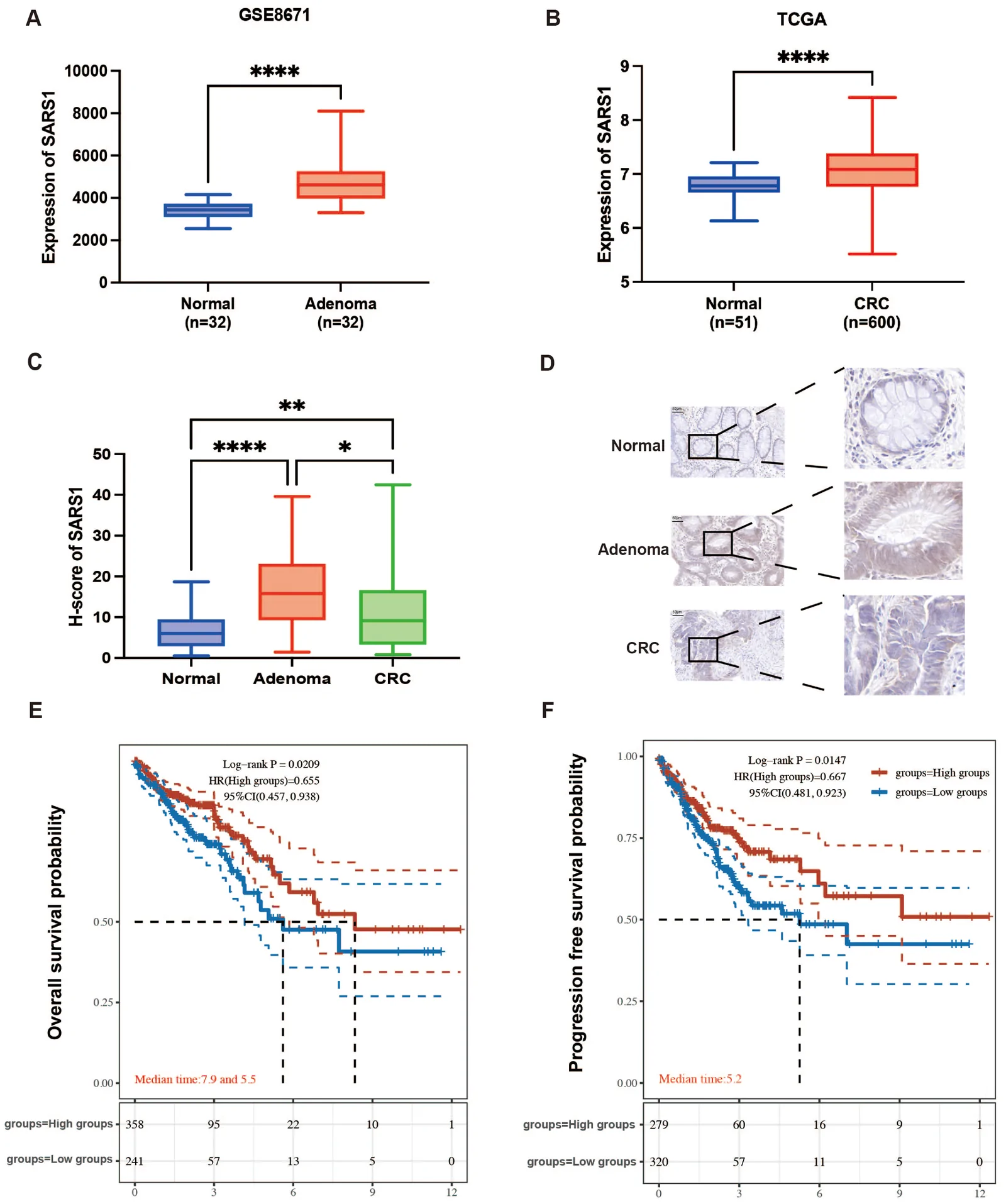
The analysis of SARS1 expression and its correlation with colorectal adenoma and CRC progression. (A) The box plot shows a significant upregulation of SARS1 in adenoma tissues (*n* = 32) compared to normal colorectal mucosa (*n* = 32) from the GSE8671 dataset. (B) The box plot shows a significant upregulation of SARS1 in CRC tissues (*n* = 600) compared to normal colorectal mucosa (*n* = 51) from the TCGA dataset. (C) The box plot shows that significant differences are observed between normal and adenoma tissues and between adenoma and CRC tissues based on a statistical analysis of H-scores derived from immunohistochemical staining results from 48 pairs of clinical specimens. (D) Representative images of immunohistochemical staining for SARS1 in normal, adenoma, and CRC tissues from paired clinical samples (images were captured at a magnification of 40×). (E) Association of SARS1 expression with the overall survival of patients with CRC, as illustrated by the Kaplan–Meier curve. (F) Association of SARS1 expression with the progression-free survival of patients with CRC, as illustrated by the Kaplan–Meier curve (*p < 0.05, **p < 0.01, and ****p < 0.0001).

Subsequently, we analyzed the relationship between SARS1 expression level and clinical pathological factors, as determined by the H-score statistical analysis results of 48 tissue sections and the clinical pathological factors. The results revealed that the expression level of SARS1 was significantly associated with adenoma size (*P* = 0.048; Table 1) and CRC lesion size (*P* = 0.017; Table 1).

**Table 1:** The relationship between SARS1 expression and clinical pathological factors.

***N* = 48**	**SARS1**		***P* value**
	High	Low	
**Adenoma**			
**Gender**			1
Male	19	20	
Female	5	4	
**Age**			0.558
<65	9	11	
≥65	15	13	
**Size of adenoma**			0.048
<2 cm	23	17	
≥2 cm	1	7	
**Type**			1
Tubular adenoma	17	18	
Tubulovillous adenoma	7	6	
**Pathological type**			0.221
Low-grade adenoma	18	14	
High-grade adenoma	6	10	
**Tumor**			
**Gender**			1
Male	19	20	
Female	5	4	
**Age**			0.079
<65	7	13	
≥ 65	17	11	
**Tumor size**			0.017
<5 cm	19	11	
≥5 cm	5	13	
Lymphovascular invasion			0.182
No	16	20	
Yes	8	4	
**Degree of differentiation**			0.562
II	10	12	
III	14	12	
**Tumor location**			0.776
Left colon	5	3	
Right colon	2	6	
Rectum	17	15	
**Remote metastasis**			1
No	24	24	
Yes	0	0	

Then, overall survival and progression-free survival analyses were conducted using Kaplan–Meier curves based on the TCGA datasets (consisting of 599 tumor samples). The log-rank test indicated a statistically significant difference in overall survival between the high-expression and low-expression groups (Log-rank *P* = 0.0209, Fig. 1E). The HR for the SARS1 high-expression group was 0.655 (95% CI [0.457–0.938]), suggesting that patients in the SARS1 high-expression group had a 34.5% lower hazard of death compared to those in the low-expression group. The median survival times were 7.9 years for the high-expression group and 5.5 years for the low-expression group, highlighting a survival advantage for the high-expression group in this context. The log-rank test again showed a significant difference in progression-free survival between the high-expression and low-expression groups (Log-rank *P* = 0.0147, Fig. 1F). In summary, our analysis of data from the TCGA database demonstrated that patients classified into the high-expression group had better overall survival and progression-free survival outcomes compared to those in the low-expression group. In the TCGA CRC cohort, higher SARS1 expression was associated with more favorable survival outcomes. Given that the cutoff was optimized and evaluated in the same cohort, this finding should be considered exploratory and requires independent external validation before any clinical prognostic implication can be established.

### Construction of patients-derived colorectal adenoma organoids and SARS1 downregulation in organoids

To verify the effect of reduced SARS1 expression on the proliferation of colorectal adenomas, we constructed human colorectal adenoma organoids. The constructed organoids were validated using H&E staining and IF staining. H&E staining of the adenoma specimen revealed typical adenomatous features characterized by well-defined glandular structures. The adenoma organoids exhibited similar glandular formations, confirming the successful establishment of the organoid model, mimicking the histological characteristics of the original adenoma tissue (Fig. 2A). The results of the organoid IF indicate that adenoma organoids exhibited robust Ki67 expression (indicating proliferative activity), E-cadherin positivity (confirming epithelial identity), and high c-Myc levels (validating adenomatous characteristics, Fig. 2B). Subsequently, we utilized shRNA to downregulate the expression of SARS1 in the adenoma organoids. The infected organoids were counterstained with GFP (green) to visualize the infected organoids. The images showed that the organoids maintained their structural integrity post-infection, indicating that the infection process did not disrupt their organization (Fig. 2C). Relative gene expression levels were determined by qRT-PCR, and IF staining of SARS1 was performed to evaluate the effects of shSARS1 infection. A significant downregulation of SARS1 was noted in shSARS1-infected organoids (*P* = 0.0001; Fig. 2D), as shown by qRT-PCR. In line with this, the fluorescence intensity of SARS1 decreased in the SARS1 knockdown group compared to the control group (*P* = 0.0014, Fig. 2E).

**Figure 2:**
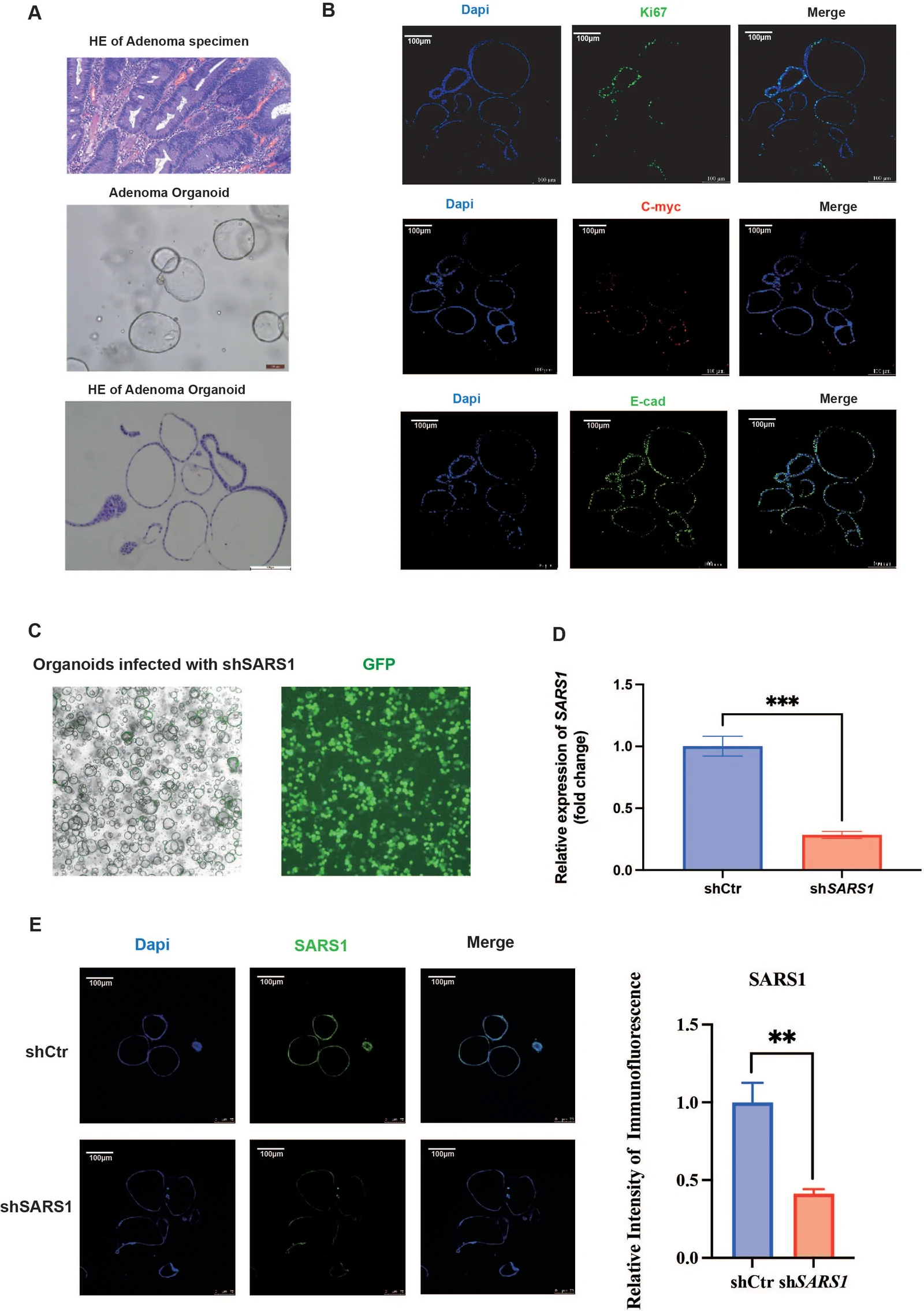
Construction and identification of human colorectal adenoma organoids and the downregulation of SARS1. (A) Representative hematoxylin and eosin (H&E) staining of adenoma tissue (40×) and adenoma organoids (20×). (B) Immunofluorescence staining of Ki67, c-Myc, and E-cadherin in adenoma organoids (images were captured at a magnification of 20×). (C) Representative images of adenoma organoids infected with shRNA (images were captured at a magnification of 4×). (D) qRT-PCR analysis of SARS1 gene expression in control (shCtrl) and SARS1 knockdown (shSARS1) adenoma organoids. (E) Immunofluorescence staining of SARS1 in control (shCtrl) and SARS1 knockdown (shSARS1) groups. (**p < 0.01 ***p < 0.001. Ki67 (mouse IgG, ZSGB-BIO, catalog #TA800648): detected with ABflo^®^ 488-conjugated Goat anti-Mouse IgG (H+L) (ABclonal, AS037)—green; C-myc (rabbit IgG, Cell Signaling Technology, catalog #5605S): detected with ABflo^®^ 555-conjugated Goat anti-Rabbit IgG (H+L) (ABclonal, AS058)—red; E-cadherin (rabbit IgG, ABclonal, catalog #A20798): detected with Goat Anti-Rabbit IgG H&L (Alexa Fluor^®^ 488) (Abcam, ab150077)—green; SARS1(Rabbit IgG, ProteinTech, catalog #15162-1-AP): detected with Goat Anti-Rabbit IgG H&L(Alexa Fluor^®^ 488), (Abcam, ab150077)—green; DAPI: nuclear counterstain (blue)).

### Knockdown of SARS1 alters proliferation-associated and stem-cell-associated markers in colorectal adenoma organoids

It has been reported that the gene expression profiles of colorectal adenocarcinoma organoids, particularly for cancer-associated markers such as c-Myc, LGR5, and OLFM4, are substantially different from those of adenoma organoids (Luo et al., 2023). To investigate how SARS1 knockdown affects proliferation-associated and stem-cell-associated markers in adenoma organoids, we examined the expression differences in c-Myc, LGR5, and OLFM4 in adenoma organoids before and after SARS1 knockdown *via* IF staining and qRT-PCR. As illustrated in Fig. 3A, the fluorescence intensity of c-Myc was significantly upregulated in the SARS1 knockdown group compared to the control group, indicating that SARS1 knockdown may enhance c-Myc expression. Conversely, the fluorescence intensity of LGR5 and OLFM4 decreased in the SARS1 knockdown group compared to the control group (Figs. 3B, 3C).

**Figure 3:**
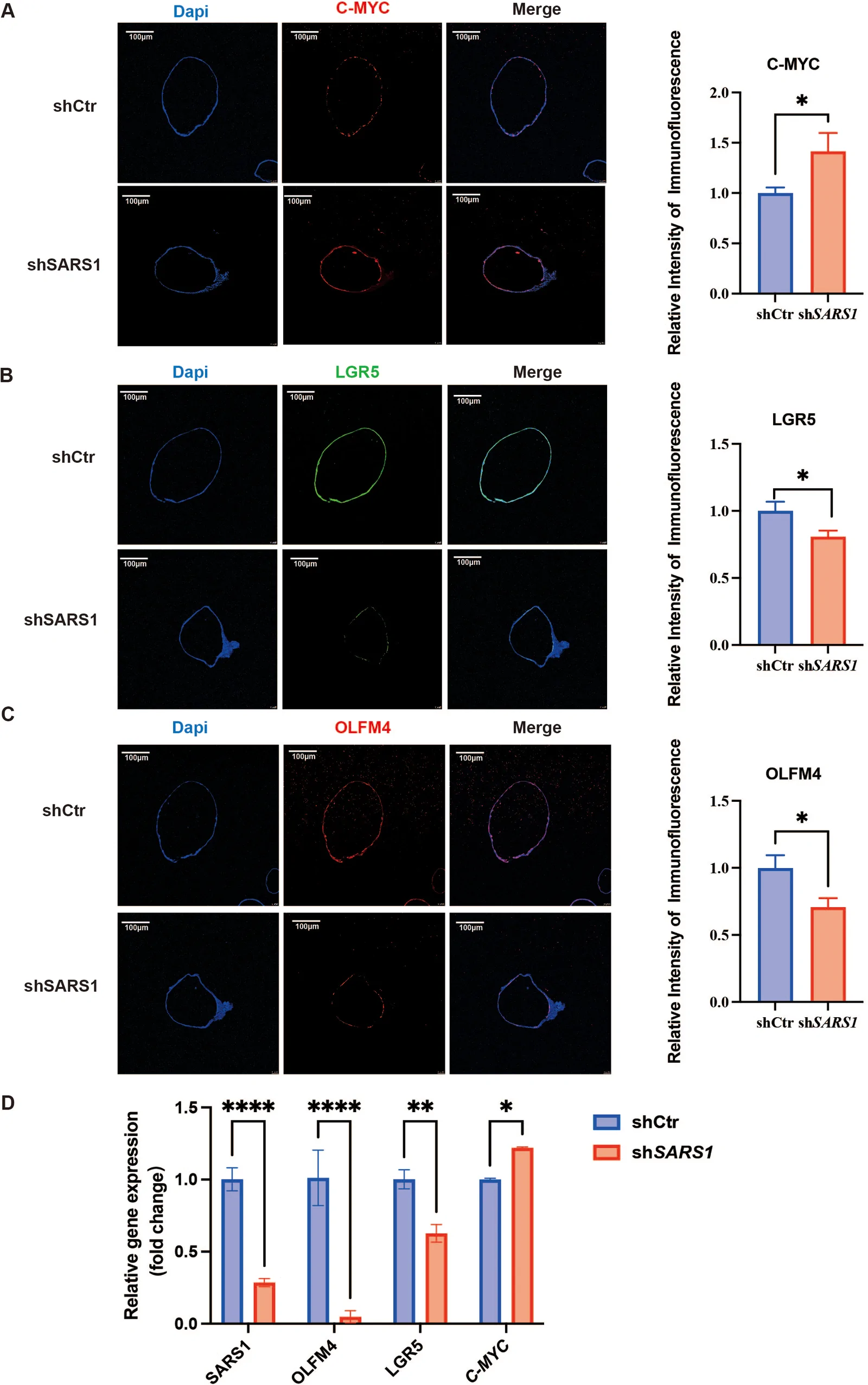
Effects of SARS1 knockdown on protein localization and gene expression in colorectal organoids. (A) Immunofluorescence staining of c-Myc in control (shCtrl) and SARS1 knockdown (shSARS1) groups. (B) Immunofluorescence staining of LGR5. (C) Immunofluorescence staining of OLFM4. (D) qRT-PCR analysis of the relative expression levels of OLFM4, LGR5, and c-Myc in the SARS1 knockdown and control groups (images were captured at a magnification of 20 ×; *p < 0.05, **p < 0.01, and ****p < 0.0001. LGR5 (mouse IgG, OriGene Tech, catalog #UM800104): detected with ABflo^®^ 488-conjugated Goat anti-Mouse IgG (H+L) (ABclonal, AS037)—green; c-Myc (rabbit IgG, Cell Signaling Technology, catalog #5605S): detected with ABflo^®^ 555-conjugated Goat anti-Rabbit IgG (H+L) (ABclonal, AS058)—red; OLFM4 (rabbit IgG, Cell Signaling Technology, catalog #39141S): detected with ABflo^®^ 555-conjugated Goat anti-Rabbit IgG (H+L) (ABclonal, AS058)—red; DAPI: nuclear counterstain (blue)).

To quantify the alterations in gene expression, we conducted qRT-PCR analysis to assess the relative expression levels of OLFM4, LGR5, and c-Myc in the SARS1 knockdown and control groups. The results, displayed in Fig. 3D, show a significant increase in c-Myc expression in the SARS1 knockdown group (*P* = 0.016), while LGR5 and OLFM4 expression was significantly reduced in the SARS1 knockdown group (*P* = 0.001 for LGR5; *P* < 0.0001 for OLFM4), aligning with the IF observations.

### The effect of SARS1 downregulation on the proliferation of human colorectal adenoma organoids

According to the analysis results in Table 1, the expression level of SARS1 is associated with the size of adenomas and colorectal cancer lesions. Moreover, the size of the tumor is an external manifestation of cell proliferation. Hence, to verify the effect of reduced SARS1 expression on the proliferation of colorectal adenomas, we monitored the morphology and density of tumor organoids after SARS1 knockdown at different time intervals. We observed that, under consistent initial plating densities, by day 9, the organoids with SARS1 knockdown were larger and more densely packed (Fig. 4A). The viability assay results indicate that on days 4 and 7, the fluorescence intensity and viability detection rate were similar between the control group and the SARS1 knockdown group. However, the fluorescence intensity significantly increased in the SARS1 knockdown group on day 9 (*P* = 0.003; Fig. 4B) and day 13 (*P* < 0.0001; Fig. 4B). Additionally, the viability detection rate also significantly increased in the SARS1 knockdown group on day 13 (*P* = 0.006; Fig. 4C). Also, we performed Ki-67 immunostaining on SARS1-knockdown organoids. As shown in Fig. 4D, the fluorescence intensity of Ki-67 was significantly upregulated in the SARS1 knockdown group compared to the control group (*P* = 0.03), suggesting that the downregulation of SARS1 increased organoid growth and metabolic viability. Therefore, we conclude that the knockdown of SARS1 promotes the growth of adenoma organoids.

**Figure 4:**
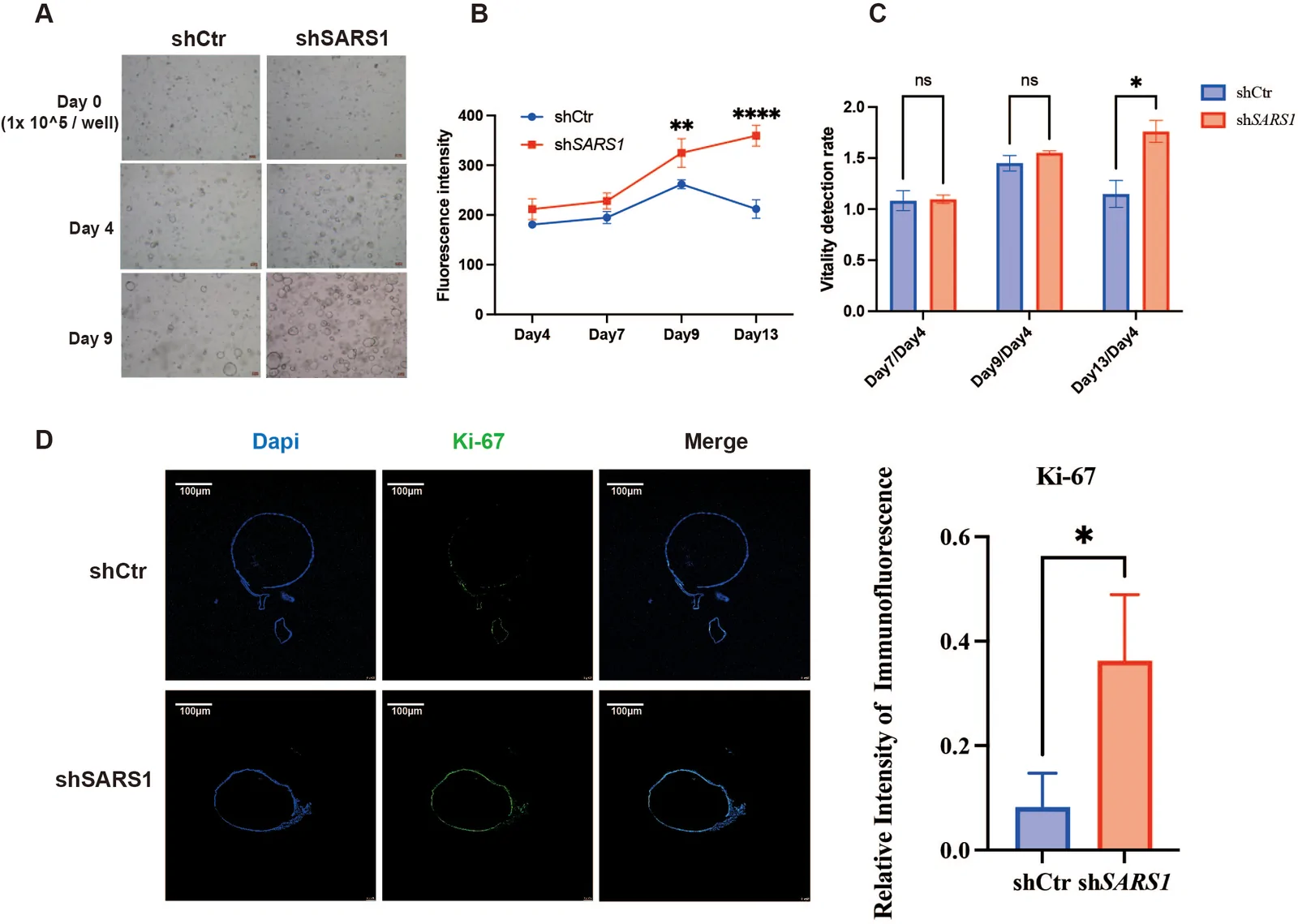
The effect of SARS1 downregulation on the proliferation of human colorectal adenoma organoids. (A) Representative images of organoids at different time points (days 0, 4, and 9) after infection with control (shCtrl) and SARS1 knockdown (shSARS1). (B) The line graph shows fluorescence intensity as a measure of organoid growth over time in control (shCtrl) and SARS1 knockdown (shSARS1) groups. (C) The bar graph displays the vitality detection rate of organoids for control (shCtrl) and SARS1 knockdown (shSARS1) groups. (D) Immunofluorescence staining of Ki-67 in control (shCtrl) and SARS1 knockdown (shSARS1) groups. (*p < 0.05**p < 0.01 and ****p < 0.0001; microscopy images were captured at a magnification of 4×; Ki-67 Immunofluorescence staining were captured at a magnification of 20 ×; Fluorescence intensity was measured with the BioGenous LivingCell-FluoTM Organoid Vitality Assay Kit (E238004, bioGenous); viable-cell metabolic reductases convert the probe into a red-fluorescent product (Ex/Em = 560/590 nm; Ki67 (mouse IgG, ZSGB-BIO, catalog #TA800648): detected with ABflo^®^ 488-conjugated Goat anti-Mouse IgG (H+L) (ABclonal, AS037)—green).

### The effect of SARS1 expression on the proliferation of CRC cells

To explore the impact of reduced SARS1 expression on the proliferation of colorectal cancer cell lines, we conducted CCK-8 assays and EdU fluorescence staining experiments. The results from qRT-PCR and WB confirmed the successful knockdown of SARS1 at both the RNA and protein levels in the HCT116 cell line (Figs. 5A, 5B). Furthermore, the CCK-8 assay results indicated that the group with a low SARS1 expression had higher optical density (OD) values (*P* = 0.015 for sh-SARS1-1; *P* = 0.028 for sh-SARS1-2; Fig. 5C), and the EdU fluorescence intensity was significantly increased (*P* = 0.007 for sh-SARS1-1; *P* = 0.004 for sh-SARS1-2; Fig. 5D).

**Figure 5:**
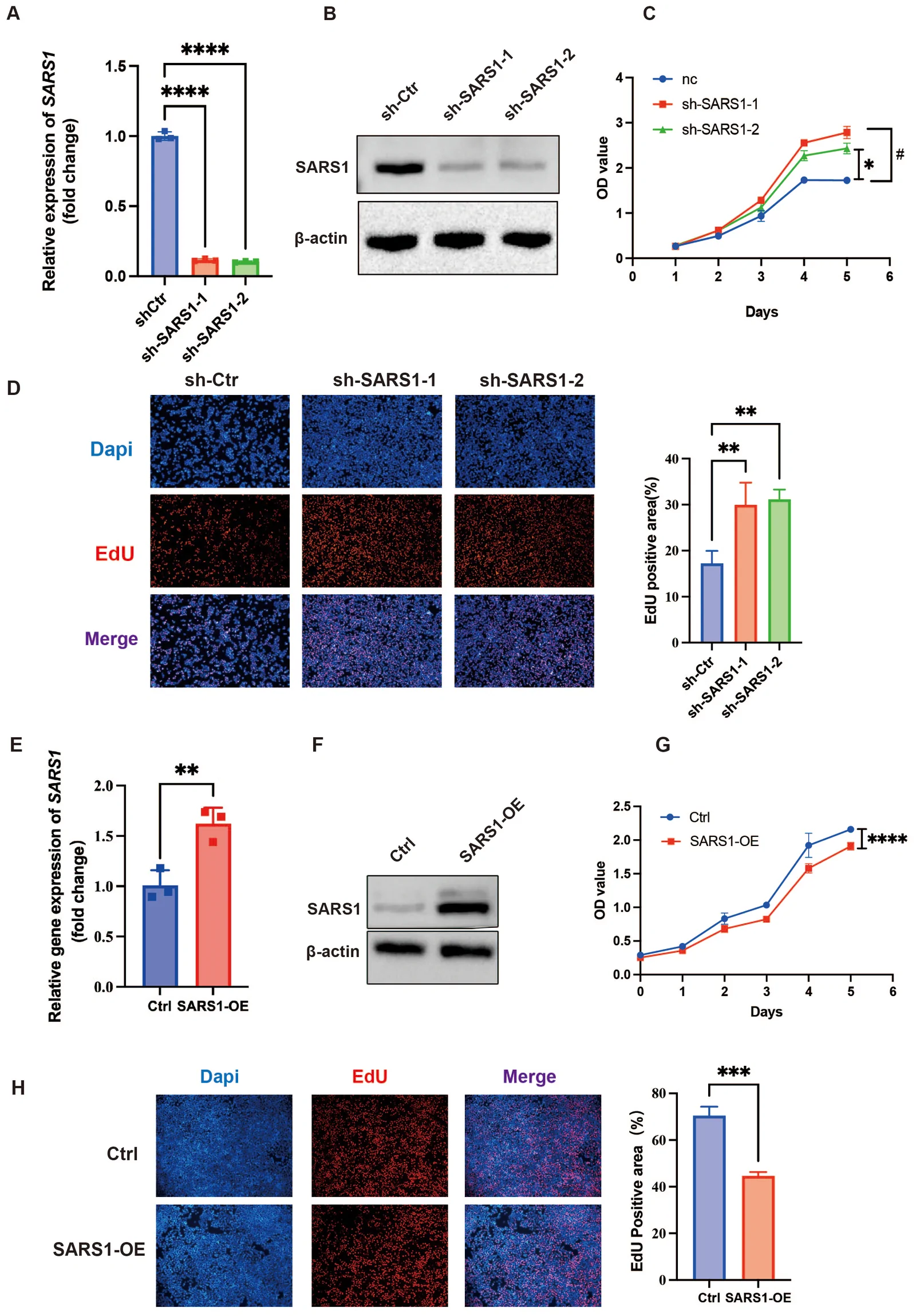
The effect of SARS1 downregulation on the proliferation of CRC cell. (A) A bar graph showing the relative mRNA expression levels of SARS1 in CRC cells after SARS1 knockdown by qRT-PCR. (B) Western Blot analysis of SARS1 expression after SARS1 knockdown. (C) A line graph representing the results of the CCK8 proliferation assay. (D) Representative images of the EdU proliferation assay and statistical analysis of the EdU-positive area for CRC cells after SARS1 knockdown. (E) A bar graph showing the relative mRNA expression levels of SARS1 in CRC cell after SARS1 over-expression by qRT-PCR. (F) Western Blot analysis of SARS1 expression after SARS1 over-expression. (G) A line graph representing the results of the CCK8 proliferation assay. (H) Representative images of the EdU proliferation assay and statistical analysis of the EdU-positive area for CRC cells after SARS1 over-expression (*, # *p* < 0.05, **p < 0.01, ***p < 0.001 and ****p < 0.0001; images were captured at a magnification of 10x. CCK-8 absorbance (450 nm, 40203ES, Yeasen). EdU-555 fluorescence (Ex/Em ≈ 555/565 nm, EdU-555 Cell-Proliferation Kit, Epizyme Biomedical Technology; nuclei were counterstained with DAPI).

To determine the consequences of SARS1 over-expression, we generated stable SARS1-over-expressing DLD-1 cells. qRT-PCR and Western blot verified elevated SARS1 expression at both the RNA and protein levels (Figs. 5E, 5F). CCK-8 assays revealed lower OD values in the over-expression group (*P* < 0.0001; Fig. 5G), and the EdU-positive area was significantly decreased (*P* = 0.0004; Fig. 5H).

Collectively, these data indicate that SARS1 down-regulation enhances colorectal-cancer cell proliferation, whereas SARS1 over-expression suppresses it. Our findings highlight SARS1 as a negative regulator of proliferation in colorectal cancer cells *in vitro*; however, these experimental results do not establish SARS1 as a universal tumor suppressor across all stages and tissues, and any therapeutic implications remain speculative pending further mechanistic and *in vivo* validation.

## Discussion

Colorectal cancer is a complex and multifactorial disease that progresses through a series of well-defined stages, from adenomas to invasive carcinomas (Xiong et al., 2025). Understanding the molecular mechanisms underlying this progression is crucial for developing effective diagnostic and therapeutic strategies (Sun et al., 2024). This study investigates the role of SARS1 in the progression of colorectal adenomas to cancer, using a combination of clinical data analysis and organoid models.

Studies have increasingly recognized the complex roles of SARS1 in tumor biology (Song et al., 2019; Li et al., 2022; Hu, 2024). As a classical aminoacyl-tRNA synthetase, SARS1 has evolved to acquire multiple non-canonical functions, playing a pivotal role in tumor angiogenesis, metabolic reprogramming, and the tumor microenvironment (Ikromov et al., 2014; Song et al., 2019). These multifaceted roles position SARS1 as a potential target for cancer therapy. For instance, SARS1 can inhibit the expression of VEGFA by competitively binding to the VEGFA promoter with c-Myc, thereby suppressing tumor angiogenesis and tumor progression (Shi et al., 2014). Additionally, research by Zhao et al. (2021) demonstrated that SARS1 regulated the expression of genes involved in lipid synthesis through its nuclear function, inhibiting lipid synthesis and proliferation in breast cancer cells. Jiang et al. (2025) further showed that the downregulation of SARS1 expression in breast cancer cells is closely associated with tumor metastasis and is mediated through the suppression of Wnt signaling pathway activation. Despite extensive research on the role of SARS1 in various cancers, its functions and mechanisms in colorectal tumors remain incompletely understood.

Our analysis of SARS1 expression in colorectal adenomas and cancers revealed that SARS1 is significantly upregulated in adenomas and cancers compared to normal colorectal mucosa. However, SARS1 expression decreases from the adenoma to cancer stages, suggesting a potential role in the early stages of tumorigenesis. The association of SARS1 expression with improved overall survival (OS) and progression-free survival (PFS) in CRC patients further underscores its clinical significance. These findings suggest that SARS1 may serve as a candidate marker reflecting adenoma-to-carcinoma progression; however, its clinical utility for early detection or prognosis requires independent validation in larger, prospective cohorts.

Additionally, studies have shown that the overall gene expression patterns of CRC organoids are distinct from those of adenoma organoids (Luo et al., 2023). In particular, the expression of c-Myc is significantly higher in CRC organoids than in adenoma organoids, while the expression of LGR5 and OLFM4 is extremely low in CRC organoids (Luo et al., 2023). In our study, we found that after SARS1 knockdown, the expression of c-Myc increased, whereas the expression of LGR5 and OLFM4 decreased, exhibiting a gene expression pattern similar to that of CRC organoids reported in previous research (Luo et al., 2023). Therefore, we hypothesize that downregulation of SARS1 may promote malignant transformation of colorectal adenomas in this experimental context, though this interpretation is based on organoid-derived observations and awaits confirmation in patient tissue series and *in vivo* models.

Our relationship analysis revealed that SARS1 expression is associated with the size of colorectal adenomas and cancers. Based on these findings, we speculate that SARS1 may influence CRC progression by modulating the proliferation of adenoma and cancer cells. Consistent with this hypothesis, our experimental data using human colorectal adenoma organoids and cancer cell lines demonstrated that SARS1 downregulation promotes the proliferation of both adenoma organoids and CRC cells. These results suggest that SARS1 functions as a negative regulator of cell proliferation in colorectal adenoma organoids and CRC cell lines, and its downregulation may contribute to cancer progression in this experimental context. These findings support a context-dependent, stage-associated role for SARS1 rather than a classical, universal tumor-suppressor identity.

It is worth noting that prior research has uniformly demonstrated that tumor suppressor genes exhibit diminished expression in cancerous tissues relative to their normal counterparts (Xiong et al., 2025). However, our investigation has uncovered a distinct pattern: although SARS1 exhibits tumor-suppressive properties in experimental systems, its expression is upregulated in colorectal cancer and adenomas compared to normal colorectal mucosa, suggesting a stage-dependent, context-specific role rather than a classical tumor suppressor. Through further literature review, we have found that there are also some genes that are highly expressed in tumors but can exert tumor-suppressive effects. The study by Liu et al. found that CXCL11 is highly expressed in CRC and is associated with anti-tumor immunity and improved prognosis (Cao et al., 2021). ARID1A, widely recognized as a tumor suppressor gene, is frequently overexpressed in primary tumors of human hepatocellular carcinoma but is conspicuously absent in metastatic lesions (Sun et al., 2017). This dichotomy implies that ARID1A may initially facilitate tumor development but is subsequently lost as the tumor progresses, suggesting a complex dual role. This study underscores the necessity of considering the stage of tumor development, dosage, and tissue context when evaluating the function of ARID1A (Sun et al., 2017). Given the complexity of gene functions in tumor progression, future research should focus on elucidating the specific regulatory mechanisms by which SARS1 exerts its effects during the progression of colorectal cancer. Similar to the ARID1A gene, when evaluating the function of SARS1, it is essential to consider the stage of tumor development, expression level, and tissue context, and future studies are needed to elucidate its precise regulatory mechanisms before therapeutic strategies can be considered.

The present study has several limitations that must be acknowledged. First, the survival analysis relied on a single public dataset (TCGA) and employed an optimal cutoff determined by the surv-cutpoint function within the same cohort. This approach is appropriate for exploratory analyses but is insufficient to support stable clinical prognostic claims without independent validation in external cohorts; consequently, all survival associations reported here should be considered preliminary. Second, we refer to SARS1 as exhibiting tumor-suppressive properties based on functional data from organoid and cell-line models; however, these experimental systems do not prove a universal tumor-suppressor identity across all stages and tissues, and the observed upregulation of SARS1 in adenomas and cancers relative to normal mucosa indicates a more nuanced, context-dependent role. Third, although we established SARS1 as a candidate marker for CRC progression, its clinical utility requires validation through large-scale retrospective analyses and prospective clinical trials to determine the optimal expression threshold for risk stratification and to evaluate its predictive value independent of conventional pathological parameters. Fourth, the use of organoid models, while powerful, may not fully recapitulate the complexity of the *in vivo* tumor microenvironment; furthermore, the number of independent patient-derived organoid lines used for each functional assay was limited, and future studies should employ larger organoid panels to confirm reproducibility. Fifth, several mechanistic questions remain unresolved: investigating SARS1 modulation in non-cancerous intestinal models will clarify whether its dysregulation represents a causative event or merely a consequence of malignant transformation; additionally, comprehensive profiling of key molecular markers during the adenoma-to-carcinoma transition—including β-catenin, KRAS mutations, and p53 status—will help delineate the precise stage at which SARS1 loss exerts its pro-tumorigenic effects and elucidate its potential interplay with other driver mutations. Finally, while the methods are detailed, the replication structure could be more transparent; we have now clarified that tissue comparisons were analyzed as paired data where applicable, and we have added the catalog numbers of key reagents to the Materials and Methods. These mechanistic investigations, though beyond the scope and resources of the current study, represent critical priorities for our ongoing research.

Moving forward, we propose a multi-pronged research agenda to address these limitations and translate our findings into clinical applications. Biobank expansion and biomarker validation: As the biobank scales up, we recommend incorporating additional stemness and differentiation markers (CD44, CD166, CD24, CD133, and EpCAM) and employing co-staining approaches to investigate the co-expression patterns of LGR5 and OLFM4 in adenoma organoids and their potential coordinated regulation by SARS1. Mechanistic elucidation: Future studies should focus on dissecting the detailed molecular mechanisms underlying SARS1-mediated tumorigenesis, particularly its context-dependent and stage-specific functions, analogous to the complex role established for ARID1A. Therapeutic exploration: The potential of SARS1 as a marker of adenoma-to-carcinoma transition warrants systematic investigation in larger clinical cohorts. Any therapeutic exploration of SARS1 should await a more complete understanding of its mechanistic role and validation in appropriate preclinical models. Such comprehensive approaches will not only advance our understanding of SARS1 biology but also facilitate its clinical translation for early detection and targeted intervention in colorectal cancer.

## Conclusions

In conclusion, this study suggests that SARS1 exerts stage-dependent, context-specific growth-suppressive effects during the progression from colorectal adenoma to cancer, and represents a candidate marker associated with adenoma-to-carcinoma transition. The observed association between SARS1 expression and patient survival in the TCGA cohort is preliminary and requires independent validation before clinical application. Further research into the precise functional mechanisms of SARS1 across different tumor stages and tissue contexts is essential to advance our understanding of its role in colorectal cancer.

##  Supplemental Information

10.7717/peerj.21792/supp-1 Supplemental Information 1 Supplemental Tables

10.7717/peerj.21792/supp-2 Supplemental Information 2 Western Blots Weston Blots picture

10.7717/peerj.21792/supp-3 Supplemental Information 3 Raw data

10.7717/peerj.21792/supp-4 Supplemental Information 4 IHC images and data analysis

10.7717/peerj.21792/supp-5 Supplemental Information 5 Raw data of immunofluorescence images of Figure 3

10.7717/peerj.21792/supp-6 Supplemental Information 6 The immunofluorescence images of SARS1

10.7717/peerj.21792/supp-7 Supplemental Information 7 The analysis data of EdU

10.7717/peerj.21792/supp-8 Supplemental Information 8 GSE8671 Data

10.7717/peerj.21792/supp-9 Supplemental Information 9 TCGA Data

10.7717/peerj.21792/supp-10 Supplemental Information 10 Codebook for TCGA data

10.7717/peerj.21792/supp-11 Supplemental Information 11 MIQE checklist

## Additional Information and Declarations

### Data Availability

The following information was supplied regarding data availability: The data is available in the Supplemental Files. The CRAN data is available at: - https://github.com/therneau/survival
- https://cran.r-project.org/web/packages/survival/index.html, DOI: 10.32614/CRAN.package.survival
The code is available at: - Kassambara A, Kosinski M, Biecek P (2026). survminer: Drawing Survival Curves using ‘ggplot2’. R package version 0.5.2, https://kassambara.r-universe.dev/survminer. The data is available at TCGA; the complete list of accessions is available in the Supplemental Files. The raw sequence data are available at NCBI GEO: GSE8671.
