# Supplementary material for: The role of SARS1 in the progression of colorectal cancer: insights from clinical data and organoid models
Source: PeerJ. 2026 Oct 25;14:e21792. doi: 10.7717/peerj.21792 (PMC13715634; doi:10.7717/peerj.21792)
Supplement: Supplementary file 1 [file peerj-14-21792-s001.docx]

**Supplementary Table**

**Supplementary Table 1. Target sequences.**

| **Name** | **Target sequences** |
| --- | --- |
| ShRNA1 | CCCATCAGTAACGATGAGGAT（EXON4-5） |
| ShRNA2 | GCTAACCTGAAAGTCTCACAA（EXON3-4） |
| ShRNA3 | CATCAGTTTGAGAAGATTGAA（EXON7-8） |

**Supplementary Table 2. Primer sequences.**

| **Primer** | **Sequences (5'-3')** |
| --- | --- |
| Sh1-SARS1-F | AGGATCTCGAGATCCTCATCGTTACTGATGGGGAATTCTCGACCTCGAGAC |
| Sh1-SARS1-R | CGATGAGGATCTCGAGATCCTCATCGTTACTGATGGGCCGGTGTTTCGTCCTTTCCAC |
| Sh2-SARS1-F | GTCTCACAACTCGAGTTGTGAGACTTTCAGGTTAGCGAATTCTCGACCTC |
| Sh2-SARS1-R | GTCTCACAACTCGAGTTGTGAGACTTTCAGGTTAGCCCGGTGTTTCGTCCTTTCCAC |
| Sh3-SARS1-F | ATTGAACTCGAGTTCAATCTTCTCAAACTGATGGAATTCTCGACCTCGAGA |
| Sh3-SARS1-R | GAAGATTGAACTCGAGTTCAATCTTCTCAAACTGATGCCGGTGTTTCGTCCTTTCCA |

**Supplementary Table 3. Primer sequences.**

| **Name of primer** | **Sequences (5'-3')** | |
| --- | --- | --- |
| β-actin | F | CATGTACGTTGCTATCCAGGC |
|  | R | CTCCTTAATGTCACGCACGAT |
| SARS1 | F | CAGCCCTCATCCGAGAGAC |
|  | R | TCTGCCCGAAATCTACATCGT |
| c-Myc | F | GGCTCCTGGCAAAAGGTCA |
|  | R | CTGCGTAGTTGTGCTGATGT |
| LGR5 | F | GAGTTACGTCTTGCGGGAAAC |
|  | R | TGGGTACGTGTCTTAGCTGATTA |
| OLFM4 | F | ACTGTCCGAATTGACATCATGG |
|  | R | TTCTGAGCTTCCACCAAAACTC |

**Supplementary Table 4. Antibody**

| Primary antibody species | Primary antibody name | Catalog number | Manufacturer | Corresponding secondary antibody name | Catalog number | Manufacturer |
| --- | --- | --- | --- | --- | --- | --- |
| Rabbit IgG | E-Cadherin Antibody | A20798 | ABclonal | Goat Anti-Rabbit IgG H&L (Alexa Fluor® 488) | ab150077 | abcam |
| Mouse IgG | Anti-Ki67 Antibody | TA800648 | ZSGB-BIO | ABflo® 488-conjugated Goat anti-Mouse IgG (H+L) | AS037 | ABclonal |
| Mouse IgG | LGR5 Antibody | UM800104 | OriGene Tech | ABflo® 488-conjugated Goat anti-Mouse IgG (H+L) | AS037 | ABclonal |
| Rabbit IgG | c-Myc Antibody | 5605S | CellSignalingTechnology | ABflo® 555-conjugated Goat anti-Rabbit IgG (H+L) | AS058 | ABclonal |
| Rabbit IgG | Olfm4 Antibody | 39141S | CellSignalingTechnology | ABflo® 555-conjugated Goat anti-Rabbit IgG (H+L) | AS058 | ABclonal |
