# Supplementary material for: The role of SARS1 in the progression of colorectal cancer: insights from clinical data and organoid models
Source: PeerJ. 2026 Oct 25;14:e21792. doi: 10.7717/peerj.21792 (PMC13715634; doi:10.7717/peerj.21792)
Supplement: Supplementary file 2 [file peerj-14-21792-s010.docx]

**Codebook for TCGA.xlsx**

| Variable | Type | Definition |
| --- | --- | --- |
| ID | Character | TCGA sample barcode  |
| time | Continuous | Follow-up time from diagnosis, in years (range 0–12.3) |
| Staus | Binary | Survival status: 0 = censored (patient alive, or no death observed at last follow-up); 1 = event (death occurred or progression) |
| RS | Continuous | Risk score derived from the SARS1-based prognostic model |
| Label | Categorical | Risk group obtained by dichotomizing RS at a cutoff: "Low groups" = low-risk group; "High groups" = high-risk group |

Sheet "TCGA PFS": identical variable definitions, except that Staus = 1 denotes the composite event of tumor progression and Staus = 0 denotes censoring free of progression.

**Sheet "SARS1 expression on TCGA":**

| Variable | Type | Definition |
| --- | --- | --- |
| Group | Categorical | "G1 (n = 600)" = tumor tissue; "Normal (n = 51)" = normal tissue |
| Type | Character | Gene symbol (SARS1) |
| values | Continuous | SARS1 mRNA expression level |
