# Supplementary material for: The role of SARS1 in the progression of colorectal cancer: insights from clinical data and organoid models
Source: PeerJ. 2026 Oct 25;14:e21792. doi: 10.7717/peerj.21792 (PMC13715634; doi:10.7717/peerj.21792)
Supplement: Supplementary file 4 [file peerj-14-21792-s004.zip › supplementary file 3/supplementary file 3-SERS IHC-PIC.docx]

N: normal	A: adenoma T: cancer

# IHC Picture

## 1N

## 1A

## 1T

## 2N

## 2A

## 2T

## 3N

## 3A

## 3T

## 4N

## 4A

## 4T

## 5N

## 5A

## 5T

## 6N

## 6A

## 6T

## 7N

## 7A

## 7T

## 8N

## 8A

## 8T

## 9N

## 9A

## 9T

## 10N

## 10A

## 10T

## 11N

## 11A

## 11T

## 12N

## 12A

## 12T

## 13N

## 13A

## 13T

## 14N

## 14A

## 14T

## 15N

## 15A

## 15T

## 16N

## 16A

## 16T

## 17N

## 17A

## 17T

## 18N

## 18A

## 18T

## 19N

## 19A

## 19T

## 20N

## 20A

## 20T

## 21N

## 21A

## 21T

## 22N

## 22A

## 22T

## 23N

## 23A

## 23T

## 24N

## 24A

## 24T

## 25N

## 25A

## 25T

## 26N

## 26A

## 26T

## 27N

## 27A

## 27T

## 28N

## 28A

## 28T

## 29N

## 29A

## 29T

## 30N

## 30A

## 30T

## 31N

## 31A

## 31T

## 32N

## 32A

## 32T

## 33N

## 33A

## 33T

## 34N

## 34A

## 34T

## 35N

## 35A

## 35T

## 36N

## 36A

## 36T

## 37N

## 37A

## 37T

## 38N

## 38A

## 38T

## 39N

## 39A

## 39T

## 40N

## 40A

## 40T

## 41N

## 41A

## 41T

## 42N

## 42A

## 42T

## 43N

## 43A

## 43T

## 44N

## 44A

## 44T

## 45N

## 45A

## 45T

## 46N

## 46A

## 46T

## 47N

## 47A

## 47T

## 48N

## 48A

## 48T
