# Supplementary material for: The role of SARS1 in the progression of colorectal cancer: insights from clinical data and organoid models
Source: PeerJ. 2026 Oct 25;14:e21792. doi: 10.7717/peerj.21792 (PMC13715634; doi:10.7717/peerj.21792)
Supplement: Supplementary file 4 [file peerj-14-21792-s004.zip › supplementary file 3/H-score.docx]

# H-score

N: normal	A: adenoma T: cancer

| (Names of Images) | Positive Area, % | Mean Density | Area Density | H-Score |
| --- | --- | --- | --- | --- |
| 1N | 4.97% | 0.1217 | 0.006046 | 6.8482 |
| 1A | 10.07% | 0.1201 | 0.012091 | 13.5358 |
| 1T | 12.13% | 0.1173 | 0.014226 | 16.9790 |
| 2N | 1.91% | 0.1332 | 0.002541 | 2.7443 |
| 2A | 6.78% | 0.1438 | 0.009753 | 10.1813 |
| 2T | 2.10% | 0.0980 | 0.002057 | 2.4356 |
| 3N | 0.85% | 0.1113 | 0.000944 | 1.0338 |
| 3A | 1.20% | 0.1085 | 0.001301 | 1.4434 |
| 3T | 2.02% | 0.1102 | 0.002222 | 2.4824 |
| 4N | 0.31% | 0.1466 | 0.000458 | 0.5465 |
| 4A | 7.22% | 0.1483 | 0.010698 | 11.2849 |
| 4T | 2.45% | 0.1231 | 0.003017 | 3.2013 |
| 5N | 4.49% | 0.1341 | 0.006021 | 6.3119 |
| 5A | 12.73% | 0.1904 | 0.024226 | 23.2486 |
| 5T | 15.61% | 0.1407 | 0.021964 | 23.3149 |
| 6N | 6.57% | 0.1319 | 0.008665 | 9.2436 |
| 6A | 16.54% | 0.1492 | 0.024672 | 26.5263 |
| 6T | 5.16% | 0.1163 | 0.006006 | 6.4147 |
| 7N | 2.34% | 0.1219 | 0.002855 | 3.1272 |
| 7A | 2.55% | 0.1316 | 0.003354 | 3.5792 |
| 7T | 8.60% | 0.1647 | 0.014164 | 14.4319 |
| 8N | 2.74% | 0.1308 | 0.003586 | 3.9424 |
| 8A | 10.19% | 0.1575 | 0.016057 | 16.9234 |
| 8T | 9.75% | 0.1385 | 0.013502 | 14.8794 |
| 9N | 9.54% | 0.1377 | 0.013138 | 14.2955 |
| 9A | 8.19% | 0.1463 | 0.011984 | 12.5608 |
| 9T | 30.25% | 0.0875 | 0.026464 | 42.4612 |
| 10N | 3.41% | 0.1396 | 0.004765 | 4.9673 |
| 10A | 11.43% | 0.1234 | 0.014109 | 16.8792 |
| 10T | 1.62% | 0.1154 | 0.001865 | 2.0405 |
| 11N | 3.52% | 0.1337 | 0.004706 | 4.9976 |
| 11A | 16.88% | 0.1160 | 0.019579 | 23.7352 |
| 11T | 4.66% | 0.1497 | 0.006978 | 7.4984 |
| 12N | 3.35% | 0.1417 | 0.004751 | 4.9985 |
| 12A | 4.24% | 0.1534 | 0.006497 | 6.8743 |
| 12T | 1.38% | 0.1839 | 0.002542 | 2.4607 |
| 13N | 7.04% | 0.1522 | 0.010717 | 10.8974 |
| 13A | 14.82% | 0.1433 | 0.021230 | 22.6058 |
| 13T | 14.52% | 0.1397 | 0.020286 | 23.4195 |
| 14N | 9.54% | 0.1616 | 0.015411 | 15.4117 |
| 14A | 12.61% | 0.1634 | 0.020593 | 21.4616 |
| 14T | 13.44% | 0.1387 | 0.018642 | 19.9934 |
| 15N | 13.74% | 0.1215 | 0.016693 | 18.6937 |
| 15A | 16.28% | 0.1316 | 0.021428 | 23.4827 |
| 15T | 6.97% | 0.1266 | 0.008821 | 9.5936 |
| 16N | 6.90% | 0.1350 | 0.009319 | 9.9782 |
| 16A | 12.47% | 0.1554 | 0.019366 | 20.1929 |
| 16T | 2.57% | 0.1302 | 0.003342 | 3.4627 |
| 17N | 7.12% | 0.1282 | 0.009128 | 10.0362 |
| 17A | 8.49% | 0.1446 | 0.012268 | 13.0366 |
| 17T | 12.02% | 0.1663 | 0.019987 | 20.6417 |
| 18N | 1.34% | 0.1909 | 0.002553 | 2.3802 |
| 18A | 14.15% | 0.1601 | 0.022656 | 23.4591 |
| 18T | 22.98% | 0.1418 | 0.032589 | 35.7075 |
| 19N | 11.29% | 0.1449 | 0.016356 | 17.3475 |
| 19A | 12.04% | 0.1659 | 0.019972 | 20.0816 |
| 19T | 20.13% | 0.1327 | 0.026709 | 31.8065 |
| 20N | 4.30% | 0.1518 | 0.006524 | 6.6560 |
| 20A | 10.28% | 0.1807 | 0.018574 | 18.3740 |
| 20T | 4.57% | 0.1405 | 0.006426 | 6.7704 |
| 21N | 10.16% | 0.1510 | 0.015343 | 15.8529 |
| 21A | 3.93% | 0.1250 | 0.004908 | 5.2358 |
| 21T | 4.27% | 0.1276 | 0.005444 | 5.7111 |
| 22N | 5.00% | 0.1229 | 0.006140 | 6.5575 |
| 22A | 6.02% | 0.1447 | 0.008702 | 9.0508 |
| 22T | 0.97% | 0.1465 | 0.001421 | 1.4367 |
| 23N | 1.06% | 0.1411 | 0.001490 | 1.5226 |
| 23A | 3.16% | 0.1323 | 0.004184 | 4.5926 |
| 23T | 13.27% | 0.1261 | 0.016734 | 18.1902 |
| 24N | 6.01% | 0.2662 | 0.015995 | 12.0077 |
| 24A | 23.26% | 0.1524 | 0.035456 | 37.1038 |
| 24T | 10.91% | 0.1289 | 0.014064 | 15.5844 |
| 25N | 8.64% | 0.1425 | 0.012314 | 12.8923 |
| 25A | 19.10% | 0.1779 | 0.033985 | 33.9787 |
| 25T | 0.60% | 0.1358 | 0.000819 | 0.8233 |
| 26N | 5.24% | 0.1567 | 0.008207 | 8.2425 |
| 26A | 22.58% | 0.1669 | 0.037688 | 39.6208 |
| 26T | 8.68% | 0.1557 | 0.013517 | 14.4331 |
| 27N | 6.82% | 0.1518 | 0.010350 | 10.6196 |
| 27A | 9.15% | 0.1575 | 0.014410 | 15.1300 |
| 27T | 2.74% | 0.1320 | 0.003615 | 4.0623 |
| 28N | 1.49% | 0.1217 | 0.001817 | 1.9082 |
| 28A | 10.12% | 0.1515 | 0.015338 | 16.1180 |
| 28T | 15.59% | 0.1760 | 0.027452 | 27.9324 |
| 29N | 3.01% | 0.1522 | 0.004583 | 4.7186 |
| 29A | 13.84% | 0.1659 | 0.022961 | 23.9266 |
| 29T | 6.20% | 0.1362 | 0.008441 | 8.9484 |
| 30N | 4.84% | 0.1375 | 0.006656 | 7.0794 |
| 30A | 26.46% | 0.1312 | 0.034726 | 38.6063 |
| 30T | 6.67% | 0.1250 | 0.008336 | 9.0305 |
| 31N | 5.38% | 0.1472 | 0.007915 | 8.2355 |
| 31A | 15.94% | 0.1477 | 0.023548 | 24.9608 |
| 31T | 9.73% | 0.1312 | 0.012763 | 14.2176 |
| 32N | 1.83% | 0.1483 | 0.002707 | 2.7955 |
| 32A | 2.17% | 0.1477 | 0.003208 | 3.2999 |
| 32T | 3.89% | 0.1288 | 0.005006 | 5.3310 |
| 33N | 1.82% | 0.1367 | 0.002494 | 2.6082 |
| 33A | 2.12% | 0.1560 | 0.003303 | 3.4245 |
| 33T | 4.21% | 0.0990 | 0.004170 | 5.4081 |
| 34N | 6.21% | 0.1389 | 0.008628 | 9.5449 |
| 34A | 6.05% | 0.1698 | 0.010282 | 10.3001 |
| 34T | 6.19% | 0.1331 | 0.008248 | 9.2260 |
| 35N | 2.77% | 0.1348 | 0.003729 | 4.0631 |
| 35A | 16.65% | 0.1668 | 0.027771 | 28.7162 |
| 35T | 6.51% | 0.1349 | 0.008783 | 9.3145 |
| 36N | 4.23% | 0.1527 | 0.006455 | 6.6024 |
| 36A | 9.70% | 0.1525 | 0.014796 | 15.8781 |
| 36T | 5.99% | 0.1193 | 0.007149 | 8.0950 |
| 37N | 5.55% | 0.0727 | 0.004038 | 5.7240 |
| 37A | 12.87% | 0.0773 | 0.009949 | 13.4425 |
| 37T | 1.35% | 0.1045 | 0.001406 | 1.5322 |
| 38N | 0.35% | 0.1686 | 0.000586 | 0.5362 |
| 38A | 1.49% | 0.1251 | 0.001865 | 1.9717 |
| 38T | 9.53% | 0.1051 | 0.010014 | 11.9748 |
| 39N | 4.48% | 0.1446 | 0.006471 | 6.7796 |
| 39A | 9.23% | 0.1680 | 0.015503 | 15.6157 |
| 39T | 10.14% | 0.1040 | 0.010544 | 12.0213 |
| 40N | 2.48% | 0.1357 | 0.003360 | 3.5779 |
| 40A | 10.66% | 0.1645 | 0.017543 | 17.9783 |
| 40T | 18.06% | 0.1541 | 0.027830 | 30.5194 |
| 41N | 4.34% | 0.1557 | 0.006750 | 6.7607 |
| 41A | 14.64% | 0.1413 | 0.020695 | 22.1518 |
| 41T | 16.13% | 0.1110 | 0.017913 | 21.9178 |
| 42N | 5.04% | 0.1302 | 0.006562 | 7.1503 |
| 42A | 8.36% | 0.1274 | 0.010646 | 11.2991 |
| 42T | 8.85% | 0.0879 | 0.007778 | 9.7046 |
| 43N | 3.51% | 0.1181 | 0.004142 | 4.6205 |
| 43A | 1.74% | 0.1371 | 0.002389 | 2.5515 |
| 43T | 2.00% | 0.1265 | 0.002532 | 2.6338 |
| 44N | 2.90% | 0.1115 | 0.003231 | 3.6521 |
| 44A | 11.25% | 0.1287 | 0.014478 | 15.7733 |
| 44T | 1.42% | 0.1393 | 0.001980 | 2.0263 |
| 45N | 2.49% | 0.1277 | 0.003180 | 3.4503 |
| 45A | 14.75% | 0.1421 | 0.020961 | 22.6935 |
| 45T | 11.00% | 0.1289 | 0.014180 | 15.3277 |
| 46N | 0.54% | 0.1228 | 0.000663 | 0.6988 |
| 46A | 6.88% | 0.1347 | 0.009269 | 9.9542 |
| 46T | 1.90% | 0.1300 | 0.002463 | 2.5961 |
| 47N | 0.56% | 0.1229 | 0.000684 | 0.7503 |
| 47A | 2.14% | 0.1036 | 0.002215 | 2.4077 |
| 47T | 1.35% | 0.0998 | 0.001352 | 1.5220 |
| 48N | 1.48% | 0.1090 | 0.001616 | 1.8639 |
| 48A | 3.62% | 0.1044 | 0.003782 | 4.1659 |
| 48T | 4.62% | 0.0954 | 0.004405 | 5.3943 |
